# An Effective Color Image Encryption Based on Henon Map, Tent Chaotic Map, and Orthogonal Matrices

**DOI:** 10.3390/s22124359

**Published:** 2022-06-08

**Authors:** Shamsa Kanwal, Saba Inam, Mohamed Tahar Ben Othman, Ayesha Waqar, Muhammad Ibrahim, Fariha Nawaz, Zainab Nawaz, Habib Hamam

**Affiliations:** 1 Department of Mathematical Sciences, Faculty of Science and Technology, Fatima Jinnah Women University, The Mall, Rawalpindi 46000, Pakistan; shamsa.kanwal@fjwu.edu.pk (S.K.); saba_inam@hotmail.com (S.I.); ayeshakianee3@gmail.com (A.W.); farihasatti1996@gmail.com (F.N.); zainimath1001@gmail.com (Z.N.); 2 Department of Computer Science, College of Computer, Qassim University, Buraydah 51452, Saudi Arabia; 3 Department of Information Technology, University of Haripur, Haripur 22620, Pakistan; 4 Big Data Research Center, Jeju National University, Jeju-si 63243, Korea; 5 Faculty of Engineering, Université de Moncton, Moncton, NB E1A 3E9, Canada; habib.hamam@umoncton.ca; 6 Spectrum of Knowledge Production & Skills Development, Sfax 3027, Tunisia; 7 Department of Electrical and Electronic Engineering Science, School of Electrical Engineering, University of Johannesburg, Johannesburg 2006, South Africa; 8 International Institute of Technology and Management, Commune d’Akanda, Libreville 1989, Gabon

**Keywords:** tent chaotic map, Hill cipher, orthogonal matrix, Henon map, peak signal to noise ratio (PSNR), number of pixel change rate (NPCR), unified average changing intensity (UACI), image encryption, decryption

## Abstract

In the last decade, the communication of images through the internet has increased. Due to the growing demands for data transfer through images, protection of data and safe communication is very important. For this purpose, many encryption techniques have been designed and developed. New and secured encryption schemes based on chaos theory have introduced methods for secure as well as fast communication. A modified image encryption process is proposed in this work with chaotic maps and orthogonal matrix in Hill cipher. Image encryption involves three phases. In the first phase, a chaotic Henon map is used for permuting the digital image. In the second phase, a Hill cipher is used whose encryption key is generated by an orthogonal matrix which further is produced from the equation of the plane. In the third phase, a sequence is generated by a chaotic tent map which is later XORed. Chaotic maps play an important role in the encryption process. To deal with the issues of fast and highly secured image processing, the prominent properties of non-periodical movement and non-convergence of chaotic theory play an important role. The proposed scheme is resistant to different attacks on the cipher image. Different tests have been applied to evaluate the proposed technique. The results of the tests such as key space analysis, key sensitivity analysis, and information entropy, histogram correlation of the adjacent pixels, number of pixel change rate (NPCR), peak signal to noise ratio (PSNR), and unified average changing intensity (UCAI) showed that our proposed scheme is an efficient encryption technique. The proposed approach is also compared with some state-of-the-art image encryption techniques. In the view of statistical analysis, we claim that our proposed encryption algorithm is secured.

## 1. Introduction

In the past few years, the use of digital technology has increased. Due to the frequent flow of digital data transmission over electronic media, the security of data is ultimate. Several functions, such as an armed forces database, secret cinematographic conferencing, health systems, digital payments, etc., require a fast, reliable security system to transmit data. Considering some characteristic highlights of pictures, such as mass information limit and high information repetition, the encryption of pictures is not quite the same as that of writings; consequently, it is hard to deal with them by conventional encryption strategies. Traditional ciphers such as AES [1] and DES [2] are not suitable for fast image encryption, as the ciphers consume huge computing power and high processing time. To fulfill the requirements of security of data and fast computation, many encryption techniques have been developed. Among all encryption techniques, chaotic theory-based encryption techniques are most suitable for image encryption, as they specify high speed, high security, the complexity of the process, and high computational power. Chaotic maps have several properties, including non-periodicity, sensitivity to initial conditions, and property of randomness. These are used for the confusion and diffusion process of data in image encryption. Chaotic maps boost the sanctuary of information.

Numerous encryption schemes of images have been proposed in the past years that used chaotic maps. Matthews in 1989 [3] proposed a non-linear iterative expression that tends to generate a chaotic sequence. He developed an encryption technique using chaotic logistic maps. Bourbakis and Alexopoulos [4] introduced an image encryption scheme that uses the language of SCAN for encryption in 1992. The symmetric image encryption technique was introduced in [5] by using a two-dimensional standard baker map. Scharinger [6] developed a Kolmogorov flow-based chaotic image encryption scheme that uses a register shift pseudo-random generator, in which permutation is performed through a controlled key chaotic system by taking the whole image as a single block. Yen and Guo [7] introduced an encryption technique named BRIE that is based on the chaotic logistic map. The encryption technique BRIE works by recirculating the pixels bitwise. The BRIE secret key contains an initial condition of the chaotic logistic map and two integers. Yen and Guo [8] introduced an encryption technique named CKBA (Chaotic Key Based Algorithm) that works in a way in which a binary sequence is considered as a key that is generated by using a chaotic system. The image pixels are rearranged according to the created parallel arrangement and afterward XORed and XNORed with the chosen key. Recently, Li [9] has introduced a video encryption scheme known as CVES (Chaotic Video Encryption Scheme) based on multiple digital chaotic systems. Pseudo-random signals are generated from 2*n* chaotic maps to cover the video and to execute pseudo-random permutation of the hidden video.

The current work is encouraged by the theme and functions used in the existing literature. A novel image encryption technique has been introduced in this work by combining Henon map and tent logistic maps with Hill cipher which exhibits tight security. The proposed scheme uses chaotic maps to generate a sequence for permutations and bitwise XOR and Hill cipher for the substitution phase. For higher security levels, the key for Hill cipher is generated by the orthogonal matrix from the equation of a plane. The proposed scheme is executed and experimented with considering color images. Security tests such as information entropy, UACI, PSNR, correlation factors analysis, and NPCR are used to assess and evaluate the performance of the proposed approach. The proposed approach is compared with the state-of-the-art approaches.

The rest of the paper is outlined as follows: Section 2 consists of the mathematical preliminaries. Section 3 describes the process of image encryption and decryption algorithms. Section 4 gives the specification of implementation results and performance evaluation of encryption and decryption algorithms. Section 5 summarizes the whole work of the presented scheme.

## 2. Mathematical Preliminaries

Our proposed scheme is composed of the following mathematical concepts: Henon map, orthogonal matrix, and chaotic tent map. Chaotic maps are simple maps that are sensitive to their starting conditions. A minor change in the values of starting conditions can alter the results at a large scale.

### 2.1. Henon Map

The Henon map was introduced by Michel Henon in 1969. It is a discrete dynamic map that exhibits chaotic behavior, as it is sensitive to its initial conditions. It is defined as:

(1) $$X\left(n+1\right)=1-aX{\left(n\right)}^{2}+Y\left(n\right)$$

(2) Y(n+1)=b(X(n)).

The vigorous behavior of a chaotic system is dependent on the values of parameters a,b that are called control parameters. The parameters and conditions of the Henon map are as follows:

1. X(0), where X(0) is the initial value.
2. a∈[0,1), where a is the control parameters.
3. $K_{1}=\left(a,X\left(0\right)\right)$ is the secret key of the permutation phase

It contains many effective properties, such as Lyapunov exponent, randomness of behavior, and uniform non-variation of density variable. Due to these characteristics, the Henon map is strongly recommended for applications in the field of cryptography.

This structure is chaotic for a=1.4,X(0)=0.766,b=0.3,Y(0)=0.3432. Eventually, a small change in the values of parameters can lead to the different behavior of a system.

### 2.2. Chaotic Tent Map (CTM)

The chaotic tent map is a dynamic map with β as a real valued function. It is a piece-wise linear and continuous map having a unique maximum in the chaotic region for analyzing density and power spectrum. A chaotic tent map is defined as:

(3) $$\phi \left(n+1\right)=\{\begin{array}{l} \frac{\beta }{2}\times \phi \left(n\right);\phi \left(n\right)<0.5\\ \frac{\beta }{2}\times \left\{1-\phi \left(n\right)\right\};\phi \left(n\right)\geq 0.5 \end{array}$$

**Figure 1:**
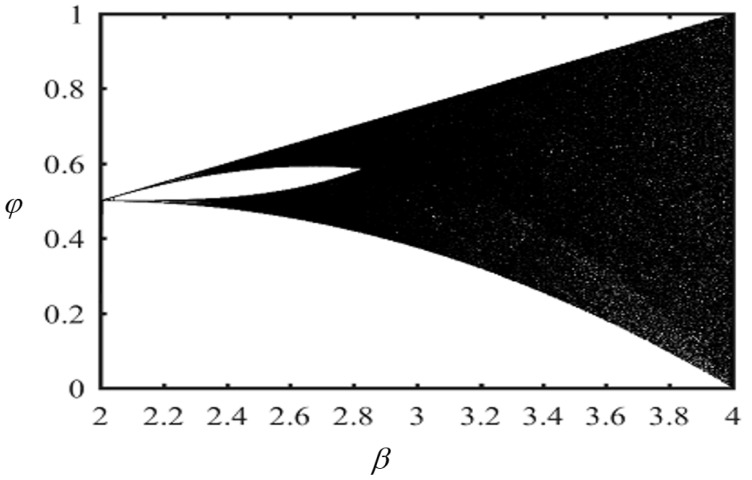
Bifurcation diagram of chaotic tent map.

The conditions and the parameter of CTM are ϕ(0)∈(0, 1), which is the initial condition, and β∈(0, 4), where β is the control bifurcation parameter. We have used the values ϕ(0)=0.66 and β=3.78. Figure 1 shows the bifurcation diagram of a chaotic tent map.

As shown in Figure 1, by analyzing the dynamic behavior of CTM, it is noted that it has a good enough range of chaos. When the bifurcation phenomenon occurs, the system is indeed chaotic. Due to its sensitivity to initial value, intrinsic randomness, and a good chaotic parameter interval, the CTM is used for developing chaotic image encryption algorithms.

### 2.3. Orthogonal Matrix

A matrix A is said to be orthogonal if A has the following property:

$$A^{t}A=I\Rightarrow A^{t}=A^{-1},$$

where $A^{t}$ is the transpose of A, and I is the identity matrix.

## 3. Image Encryption and Decryption Algorithms

**Figure 2:**
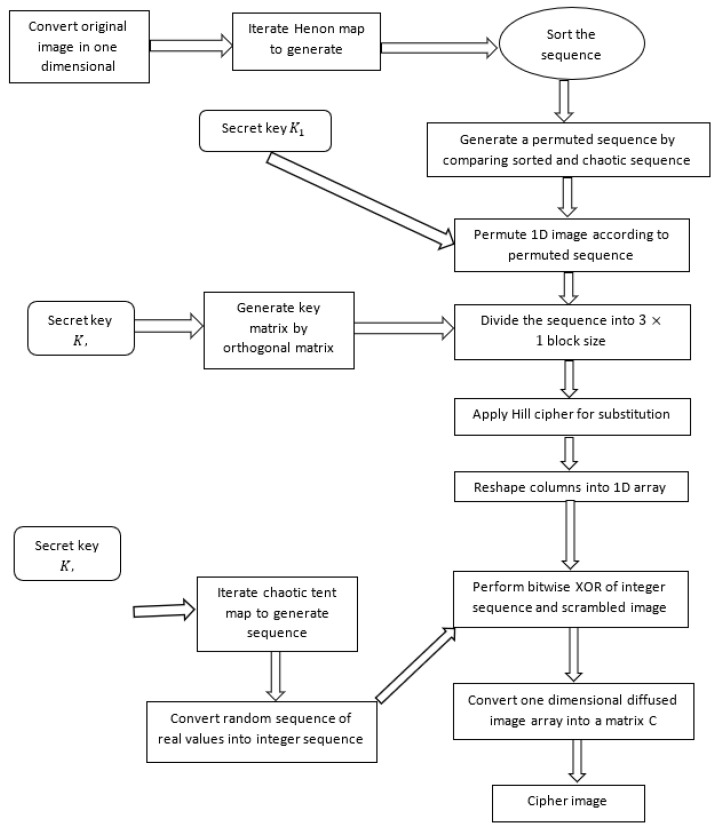
Workflow of proposed encryption scheme.

The whole scheme of image encryption consists of three phases. The first phase uses the Henon map to generate a sequence for permuting the pixels of an image. In the second phase, the permuted pixels are multiplied with the key invertible matrix, which is produced by a secret orthogonal matrix. The last phase consists of a process of confusion in such a way that a new sequence which is generated from a new chaotic tent map is XORed with previously generated results. The complexity of the scheme helps in resisting attacks from the attackers. Figure 2 depicts the workflow of our proposed encryption technique.

### 3.1. Permutation Process

The permutation phase of our proposed cryptosystem consists of permuting the positions of the pixels of an original image as shown in Algorithm 1. In the first phase of our scheme, to permute the pixels’ positions, the Henon map is used with the key $K_{1}$. By using $K_{1}$, the Henon map is reiterated to produce a sequence. The produced chaotic sequence is arranged in ascending order. The permuted sequence is obtained by comparing the arrangements of chaotic and sorted sequences. The one-dimensional array of the original image is obtained by using the permuted sequence.
**Algorithm 1.** Pixel Permutation**Input:** Secret key $K_{1}=\left(a,X\left(0\right)\right)$ Henon map (1), Color image I.
**Output:** Array *L* of permuted pixels of an image I.
  1.Take the original image *I*, which is stored in an array *Y* with size *M* = *P* × *Q* × 3, where *P* indicates the number of rows and *Q* indicates the number of columns of the image matrix *I*.
  2.$\mathrm{Use}\;\mathrm{the}\;\mathrm{key}\;K_{1}$$\;\mathrm{with}\;\mathrm{Henon}\;\mathrm{map}\;(1)\;\mathrm{to}\;\mathrm{produce}\;a\;\mathrm{sequence}.\;\mathrm{Generate}\;\mathrm{the}\;\mathrm{chaotic}\;\mathrm{sequence}\;H=\left\{h_{1},h_{2},\ldots ,h_{M}\right\}$$\;\mathrm{and}\;\mathrm{sort}\;\mathrm{it}\;\mathrm{in}\;\mathrm{ascending}\;\mathrm{order},\;\mathrm{the}\;\mathrm{resulting}\;\mathrm{sequence}\;\mathrm{is}\;\underline{H}=\left\{{\underline{h}}_{1},{\underline{h}}_{2},\ldots ,{\underline{h}}_{M}\right\}$.  3.Compute the permutation vector J by noting the positions of sequence terms of H $\mathrm{in}\;\underline{H}$$\;\mathrm{and}\;\mathrm{write}\;\mathrm{down}\;\mathrm{the}\;\mathrm{transformed}\;\mathrm{positions}\;J=\left\{j_{1},j_{2},\ldots ,j_{M}\right\}.$  4.Use J to permute the positions of elements of an array Y to get L.

For the image selection, the general consideration is to take any size of P×Q×3 pixels colored image, where P and Q are the height and width, respectively. The size of the encrypted image would be the same as that of the original image.

### 3.2. Substitution Phase Using Hill Cipher with Orthogonal Matrix

The second phase is the substitution phase as shown in Algorithm 2. In this phase, the secret key $K_{2}$ is generated by the orthogonal matrix generated by an equation of a plane. The secret key $K_{2}$ is used for Hill cipher in the substitution algorithm given I Algorithm 3. The permuted image is divided into $\frac{M}{3}$ sub-blocks. These $\frac{M}{3}$ sub-blocks are one-by-one multiplied by the generated 3×3 orthogonal matrix. The result is arranged in one-dimensional array *E*.

Algorithm 2 presents the generation of a key orthogonal matrix from the equation of plane [10].
**Algorithm 2.** Key Generation of Permutation Process**Input:** Equation of plane ax+by+cz=d,a,b,c,d∈ℜ. **Output:**$\;\mathrm{Orthogonal}\;\mathrm{matrix}\;K_{2}.$   1.Let the orthogonal line O be spanned by the unit vector t$$t=\frac{\left(a,b,c\right)}{\sqrt{a^{2}+b^{2}+c^{2}}},$$$\mathrm{from}\;\mathrm{the}\;\mathrm{expression}\;Z_{w}=w-2t\langle w,t\rangle$, where 〈w,t〉 is the inner product of w and t.  2.$\mathrm{For}\;w=\left\{w_{1},\;w_{2},w_{3},\ldots ,w_{m}\right\}\in R^{m}$ be the basis of O. The basis vectors for m=3 are
$$w_{1}=\left[w_{11},w_{12},w_{13}\right]=\left[1,0,0\right]\;w_{2}=\left[w_{21},w_{22},w_{23}\right]=\left[0,1,0\right]\;w_{3}=\left[w_{31},w_{32},w_{33}\right]=\left[0,0,1\right].$$
  3.$\mathrm{The}\;\mathrm{orthogonal}\;\mathrm{key}\;\mathrm{matrix}\;K_{2}$ will be
$$K_{2}=\left[\begin{array}{ccc} z_{w11} & z_{w12} & z_{w13}\\ z_{w21} & z_{w22} & z_{w23}\\ z_{w31} & z_{w32} & z_{w33} \end{array}\right].$$



**Algorithm 3.** Hill Cipher with Orthogonal Matrix**Input:** Permuted image array L,
$K_{2}$
**Output:** An array E of order M.
  1.$\mathrm{Use}\;\mathrm{the}\;\mathrm{given}\;\mathrm{equation}\;\mathrm{of}\;a\;\mathrm{plane}\;\mathrm{to}\;\mathrm{generate}\;\mathrm{the}\;\mathrm{key}\;\mathrm{orthogonal}\;\mathrm{matrix}.\;K_{2}$ will be the orthogonal key matrix under mod 256 of order 3×3.  2.$\mathrm{Making}\;\mathrm{blocks}\;L_{r}$(i)Transform one-dimensional array into block vectors of size 3×1. The rth$\;\mathrm{block}\;\mathrm{is}\;L_{r}$, where r=1,2,3,…,M3(ii)Hill cipher is implemented by using the following formula
$$A^{r}=K_{2}\times L_{r}\left(mod256\right).$$
(iii)$\mathrm{Write}\;\mathrm{all}\;A_{r}{}^{\prime }s$ in one-dimensional array again such that E={A1,A2,A3, …,AM3}.


### 3.3. Diffusion Phase

In the last phase, the diffusion of pixels take place as shown in Algorithm 4. In this phase, by using K_3_ key, a sequence is produced by iterating a chaotic tent map (CTM) (3), and then the values of the sequence are transformed into an integer sequence by using Equation (4). The one-dimensional array is correspondingly XORed bitwise with the integer sequence. A matrix of order P×Q×3 is obtained by rearranging the one-dimensional array and from the matrix of cipher image.
**Algorithm 4.** Pixel Diffusion**Input:** The Array E$,\;\sec\mathrm{ret}\;\mathrm{key}\;K_{3}=\left(\phi \left(0\right),\beta \right)$, CTM (3) **Output:**$\;\mathrm{Encrypted}\;\mathrm{image}\;C^{\prime }$.
  1.$\mathrm{Generate}\;a\;\mathrm{sequence}\;W=\left\{W_{1},W_{2},\ldots ,W_{M}\right\}$$\;\mathrm{with}\;\mathrm{key}\;K_{3}$ and CTM (3).  2.A sequence W is transformed into an integer sequence by the given Equation (4) (4)$$P_{K}=floor\left(mod(W_{K}\times 10^{14},256\right))$$
  3.Mix each element of E $\mathrm{with}\;\mathrm{the}\;\mathrm{parallel}\;\mathrm{element}\;\mathrm{of}\;P_{K}$ and a bitwise XORing is performed to make an array $$C_{j}=P_{j}⊕E_{j}⊕C_{j-1},j=1,2,\ldots ,M.$$  4.$\mathrm{Change}\;\mathrm{the}\;\mathrm{array}\;C_{j}\;$$\mathrm{in}\;\mathrm{the}\;\mathrm{matrix}\;\mathrm{form}\;\mathrm{named}\;\mathrm{as}\;C^{\prime }$ of the size of M=P×Q×3.

### 3.4. Image Decryption Process

The process of image decryption is carried out to obtain the original image by using the reverse encryption algorithm. The proposed decryption procedure also includes three phases as shown in Algorithm 5. In the first phase, the sequence generated from the chaotic tent map (CTM) is XORed with the key $K_{3}$. The Hill cipher is used with the invertible matrix by using $K_{2}$. A random sequence is generated from the Henon map and by using key $K_{1\;}$inverse permutation is obtained. To converse the permutation, the inverse permutation is employed. The subsequent array is transformed into an image form to obtain the original image.
**Algorithm 5.** Pixel Decryption Process**Input:** Cipher image C, Secret keys $K_{1},K_{2},K_{3}$, Henon Map (1), CTM (3).
**Output:** Colored image I
  1.$\mathrm{Place}\;\mathrm{the}\;\mathrm{matrix}\;C^{\prime }$ of the cipher image in an array of order M=P×Q×3.  2.Generate a sequence W of an order M=P×Q×3$\;\mathrm{by}\;\mathrm{using}\;\mathrm{key}\;K_{3}$ and XOR it with the integer sequence generated from the relation (4).  3.$\mathrm{Each}\;\mathrm{element}\;\mathrm{of}\;C^{\prime }$ is pre-decrypted as:$$E_{j}=C_{j}⊕P_{j}⊕E_{j-1}\;j=1,2,3,\ldots ,M$$
  4.$\mathrm{Generate}\;\mathrm{an}\;\mathrm{orthogonal}\;\mathrm{matrix}\;K_{2}$ as in Algorithm 2.   5.Transform the one-dimensional array E$\;\mathrm{in}\;\mathrm{block}\;\mathrm{vectors}\;L_{r}$ of size 3×1.  6.Hill cipher is executed by using the formula $$A_{r}=K_{2}\times L_{r}\left(mod256\right)$$  7.$\mathrm{Change}\;\mathrm{all}\;A_{r}{}^{\prime }s\;$in one dimensional array L.  8.$\mathrm{Use}\;\mathrm{the}\;\sec\mathrm{ret}\;\mathrm{key}\;K_{1}$, iterate the Henon map (1) to generate a sequence H$\;\mathrm{and}\;\mathrm{obtain}\;\underline{H}\;$by cataloguing H in ascending order.  9.$\mathrm{Obtain}\;\mathrm{the}\;\mathrm{permutation}\;\mathrm{array}\;\mathrm{by}\;\mathrm{inverse}\;\mathrm{transformation}\;\mathrm{position}\;J^{-1}$.  10.$\mathrm{Using}\;J^{-1}$ on L to obtain Y  11.Rewrite Y in a matrix formation of order M to get image I

## 4. Analytical Results and Performance Evaluation

**Figure 3:**
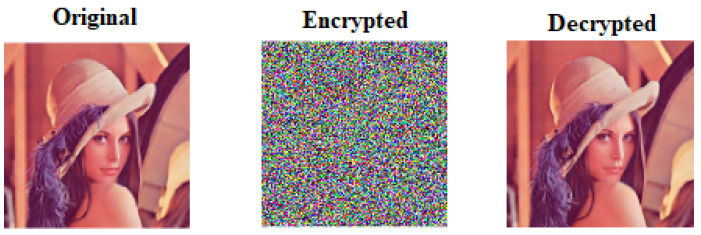
Original, encrypted, and decrypted image of Lena (256×256).

In this section, proposed algorithms are assessed by examining the statistical and differential parameters of the tests. In order to implement and evaluate our proposed encryption scheme, we have used Matlab 2018a. The sample images are downloaded from the USC-SIPI database [11]. The algorithms of permutation of pixels, mixing of the key orthogonal matrix with Hill cipher, and diffusion of a pixel are implemented to obtain the encrypted image and the original image back by using decryption algorithm. The standard colored images of Lena with pixel values of length (256 × 256), are chosen for evaluating our proposed scheme. We performed the encryption using $K_{1}=\left(0.766,\;0.3432\right),\;K_{3}=\left(0.7666,\;3.999\right)$. The sample image of Lena is chosen to compare our performance of our proposed scheme against the other chosen schemes. The input and output of the sample image Lena obtained from encryption and decryption algorithms are shown in Figure 3.

### 4.1. Statistical Analysis of Histogram

**Figure 4:**
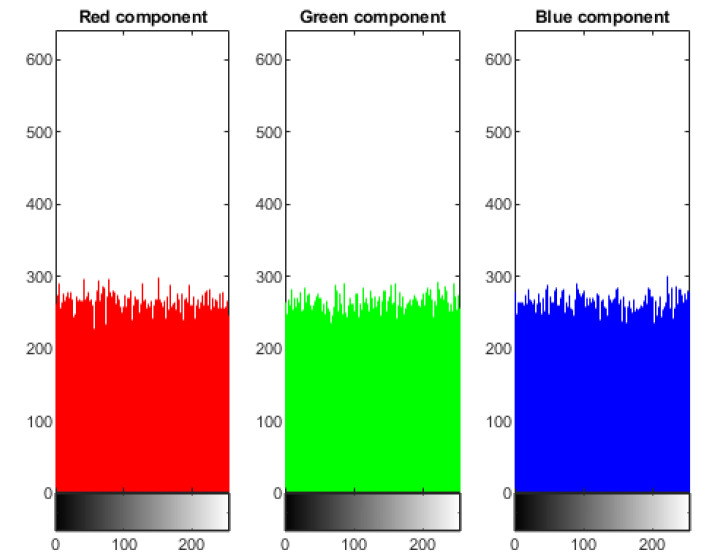
Histogram of Encrypted Image of Lena (256×256).

Analysis of histogram is the groundbreaking assessment of image pixels. It should be distinctive from the original and encoded picture. The pixels of the plain image are non-uniform and variant at every single moment. It is clearly visible that the histogram of the cipher image is fairly uniform. It is evident that no information is leaked from the cipher image of the dispersal of pixels in the original image. Figure 4 shows the three components, red, green, and blue histogram, of the coded cipher image. The histogram of cipher images is moderately uniform, as seen in Figure 4. There is no evidence about the distribution of pixels in the original image.

### 4.2. Histogram Variance Analysis

The variances of the first and encrypted picture histograms are estimated to decide the picture pixel consistency. The pictures have more noteworthy pixel consistency when the changes are more modest. It is estimated by

(5) $$var\left(X_{i}\right)=\frac{1}{r^{2}}\sum_{m=1}^{r}\sum_{n=1}^{r}\frac{{\left(x_{m}-x_{n}\right)}^{2}}{2}\;$$

where $X_{i}=\left\{x_{1},x_{2},x_{3},x_{4},\ldots ,\;x_{256}\right\}$, m and n signify the grayscale pixel esteems and $x_{m}$ and $x_{n}$ signify the number of pixels for every one of the grayscale pixel esteems m and n, individually. The suggested technique exhibits less average variance than the compared approach of [12,13], as shown in Table 1.

### 4.3. Chi-Square Test Analysis

The consistency in the histograms of the encoded pictures can likewise be advocated through chi-square test investigation. The low chi-square worth demonstrates high consistency in encoded picture histograms. It is estimated by

(6) $$\chi_{t}{}^{2}=\sum_{j=0}^{255}\frac{{\left(O_{j}-E_{j}\right)}^{2}}{E_{j}}\;$$

where the observed frequency of *j* is $O_{j}$ and the expected frequency of *j* is $E_{j}$; expected frequency is expressed as

$$E_{j}=\frac{\mathrm{image}\;\mathrm{size}}{256}$$

Table 2 illustrates that the hypothesis is accepted at both 5% and 1% levels of significance for the proposed technique. In addition, there exists uniformity of the grayscale in the histograms of encrypted images of the proposed and Refs. [12,13] algorithms. It is also depicted that the proposed scheme has a low chi-square value as compared to the Refs. [12,13] techniques, which exhibits the efficiency of our suggested method.

### 4.4. Correlation Analysis of Adjacent Pixels

**Figure 5:**
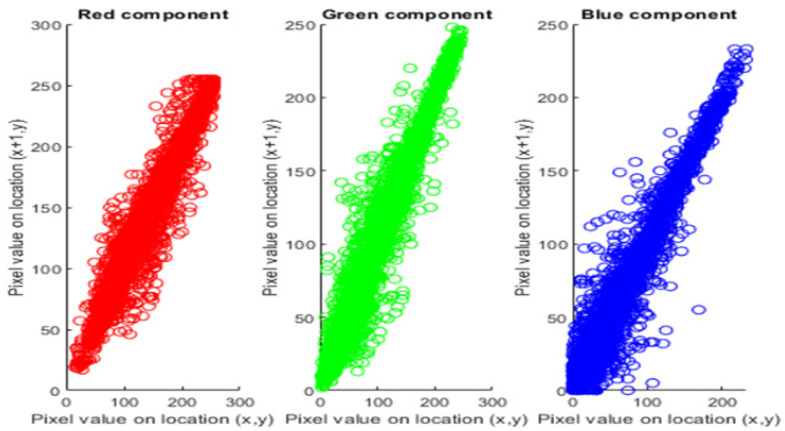
Correlation coefficient of the original color image of Lena (256 × 256 pixels).

**Figure 6:**
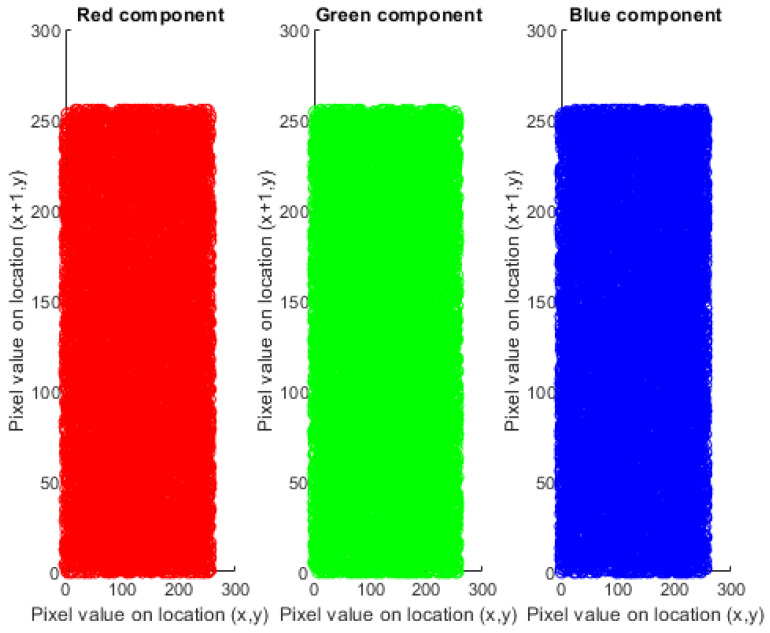
Correlation (row-wise) of the cipher image of Lena 256.

The correlation coefficient shows resemblance along the horizontal, vertical, and diagonal direction of nearby pixels. Correlation $C_{r}$ is used to test the confusion and diffusion process between the plain image and the coded image. It can be calculated by using the formula given in the Equation (7).

(7) $$C_{r}=\frac{n(\sum_{t=1}^{n}x_{t}y_{t}-\sum_{t=1}^{n}x_{t}\sum_{t=1}^{n}y_{t})}{\left(n\sum_{t=1}^{n}{\left(x_{t}\right)}^{2}-{\left(\sum_{t=1}^{n}x_{t}\right)}^{2})){\left(n\sum_{t=1}^{n}y_{t}\right)}^{2}-{\left(\sum_{t=1}^{n}y_{t}\right)}^{2}\right)}\;$$

where n is the total pixel value chosen to calculate the coefficient and $x_{t}$ and $y_{t}$ are the values of two neighboring pixels. The highest correlation factor value is 1, which shows the existence of a high correlation coefficient among the adjacent pixels. The proposed encryption technique must encrypt with low correlation coefficients which are approximately equal to zero, such that the attacker could not be able to acquire the useful data. Figure 5 and Figure 6 illustrate the distribution of the original and encrypted image pixels in RGB components.

Table 3 show the values of correlation distribution in three directions for the original and cipher image. The values show that in the cipher image the adjacent pixels are almost uncorrelated, as it is closer to zero. The number of random pixels is 16,430 pairs of pixels, and the comparison is carried out on 4500 pairs of neighboring pixels at random.

### 4.5. Mean Square Error Analysis

The mean square error (MSE) is used to measure the accuracy and variation among two images. A high value of MSE corresponds to a large difference between the ciphered and plain images. The MSE values are determined by the formulas given in expressions (8).

(8) $$\mathrm{MSE}=\frac{1}{m\times n}\sum_{i=0}^{m-1}\sum_{i=0}^{n-1}{\left(I_{P}\left(i,j\right)-I_{D}\left(i,j\right)\right)}^{2}$$

where m represents the number of rows and n represents the number of columns of the image. $I_{P}$ and $I_{D}$ represent the plain image and the cipher image, respectively. The MSE of the proposed encryption scheme of the image and its comparison with some schemes are illustrated in Table 4. It can be seen from the results that the proposed scheme has a larger MSE value than the methods suggested in Refs. [12,13]. We conclude that there is an extensive difference between plain and ciphered images in the proposed algorithm as compared to the Refs. [12,13] techniques.

### 4.6. Peak Signal to Noise Ratio Analysis

The analysis of PSNR is used to determine the quality of the ciphered image against the plain image. A low value of PSNR corresponds to a large difference between ciphered and plain image. It is analyzed by the formulas given in Equation (9).

(9) $$\mathrm{PSNR}=10\cdot log\frac{255^{2}}{MSE}$$

The value of PSNR for the proposed scheme is 8.6940. 

**Figure 7:**
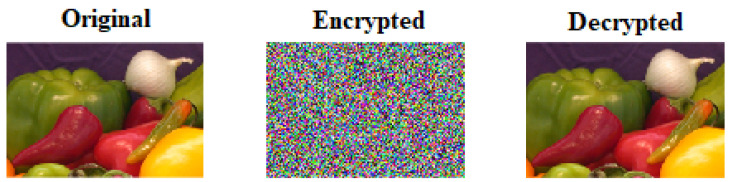
Sample image of onion (colored 198 × 135 pixels).

**Figure 8:**
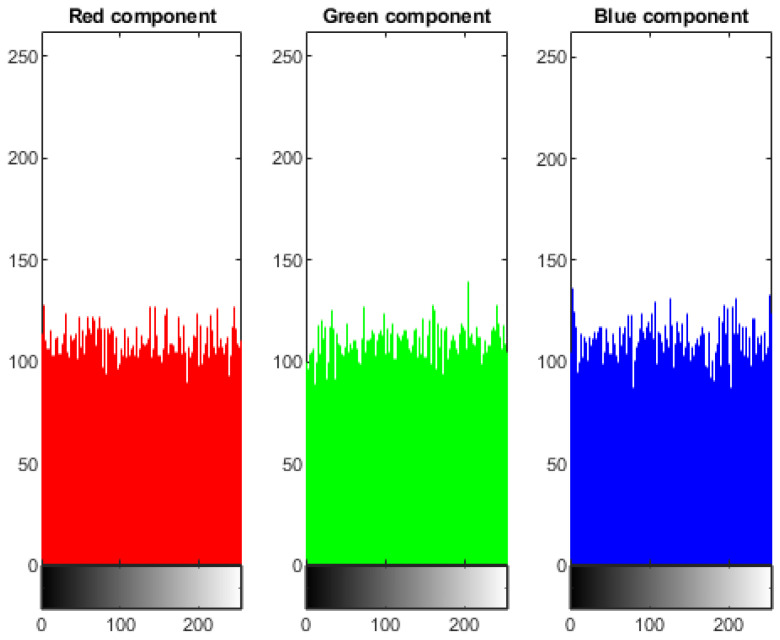
Cipher image histogram analysis of onion (colored 198 × 135 pixels).

**Figure 9:**
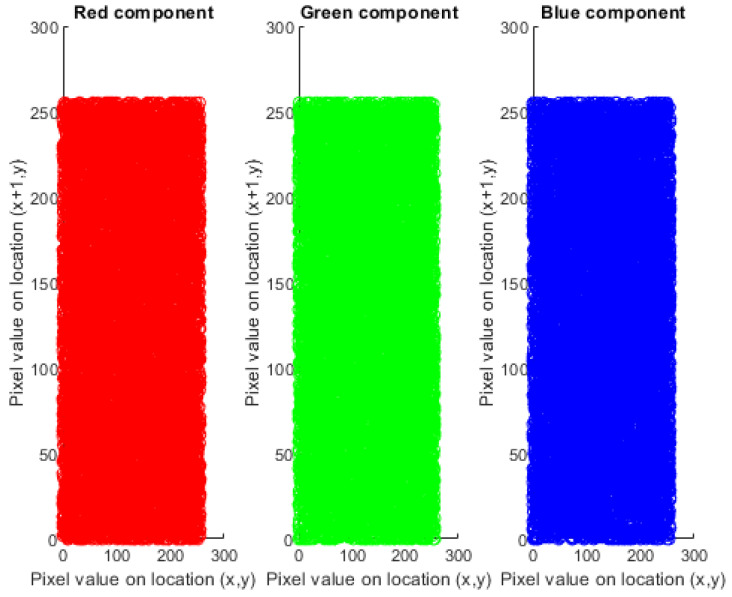
Correlation analysis of color components of onion (colored 198 × 135 pixels) cipher image.

Another sample-colored image of Onion (198 × 135 pixels) has been chosen to apply on our proposed cryptosystem. The entropy value of the onion image is calculated as 7.9975. Figure 7 shows the results of encryption and decryption of sample images. Figure 8 and Figure 9 show the histogram and correlation coefficient of plain and cipher images, respectively. Table 5 illustrates the correlation coefficient values of the sample image of the onion.

### 4.7. Sensitivity Analysis

In cryptography, plain-text sensitivity analysis is also known as differential evaluation. Two standardized tests of the number of changing pixel rate (NPCR) and the unified averaged changed intensity (UACI) are used to observe the original plain image sensitivity against external attacks. The test shows the impact whereby small variation in the plain image causes high alteration in the encrypted images. The more effective cryptosystem is designed when the higher value of NPCR is achieved and will provide security against different attacks. Both indicators can be calculated by using the formulas in Equations (10) and (11) as follows:

(10) $$\mathrm{NPCR}=\frac{\sum_{i,j}K\left(i,j\right)}{w\times h}\times 100$$

(11) $$\mathrm{UACI}=\frac{1}{w\times h}\left[\sum_{i,j}\frac{\left|X\left(i,j\right)-X{}^{\prime }\left(i,j\right)\right|}{255}\right]\times 100$$

In Equation (11), w and h represent the width and height of the cipher image, respectively. X denotes cipher image, while $X{}^{\prime }$ denotes the change of one pixel in plain image. If $X\left(i,j\right)\neq X{}^{\prime }\left(i,j\right)$, K(i,j)=1;else,K(i,j)=0. It can only be resistant to differential attacks when the values of NPCR and UACI should approach their ideal values. The ideal values of NPCR are 99.61 and UACI is 33.46. We compare the values of NPCR and UACI for the encrypted Lena image in Table 6.

It is shown that the present scheme attains peak performance for both values. In this case, the present scheme provides good resistance against “Known plain-text attack” and “Chosen plain-text attack”.

### 4.8. Information Entropy Analysis

Entropy is the measurement of an irregularity of the pixel concentrations in the cipher image. It is used to determine the entropy of information in order to measure the randomness in the cipher image. In the proposed technique, information entropy for the encrypted image *g*, which is *H*(*g*), is evaluated. The measured value of entropy of encrypted image *g* is given in Equation (12).

(12) $$H\left(g\right)=\sum_{i=0}^{255}P\left(g_{i}\right){log}_{2}\frac{1}{P\left(g_{i}\right)}$$

where $g_{i}$ is discrete pixel values and P is the probability of these values. In our example of the proposed technique of encrypted image $C^{\prime }$ with 2N as 255, the entropy value is calculated as 7.9992. Table 7 illustrates the comparison of entropy values of different encryption techniques. The calculation of entropy value illustrates that the value of entropy of our encryption algorithm is close to the standard value of entropy that is calculated with Equation (12). It confirms that no information has been lost in our proposed cryptosystem.

### 4.9. Key Space Analysis

Key space analysis is basically analysis of all the possibilities of keys used in the encryption process. The size of the key must be large enough to oppose brute force attacks. With the modern computational techniques, an algorithm can resist exhaustive attacks if the size of key space is larger than $10^{30}$ [18]. Our proposed image encryption algorithm consists of three different keys. The keys $K_{1}$ and $K_{3}$ consist of control parameters of Henon and tent maps. By observing the precision of the parameters to be $10^{-15}$, the total amount of possibilities to choose the keys could be ${\left(10^{15}\right)}^{2}\times {\left(10^{15}\right)}^{2}={\left(10\right)}^{60}\approx {\left(2\right)}^{240}$. As the size of the key of two algorithms is up to 60, our proposed permutation and confusion process is strong enough to be protected from brute force attack. Since the second key $K_{2}$ for substitution phase is generated by an equation of plane ax+by+cz=d,a,b,c,d∈ℜ, there are infinite possibilities for choosing the four coefficients of a,b,c,d. Consequently, the size of key space for $K_{2}$ is also infinite.

### 4.10. Computational Time Analysis

Consider that the quickest computer can calculate$\;2^{80}$ computations in a single second. Thus, in a single year, the wide variety of computations accomplished through the computer is $2^{80}\times 365\;\left(\mathrm{days}\right)\times 24\;\left(h\right)\times 60\;\left(\min\right)\times 60\;\left(s\right).$ As a result, the entirety of $\;2^{240}/2^{80}\times 365\times 24\times 60\times 60=10^{36}$ years is required. This time duration is enough to secure the whole cryptosystem. To face up to the brute force attack in opposition to this encryption algorithm, this computational load is large enough.

### 4.11. Key Sensitivity Analysis

The secret keys of the scheme are significant for its encryption algorithm. Our proposed encryption algorithm has three keys. In this present technique, the result of the decryption algorithm entirely changes even for a very small variation in any part of the secret key. This means that if we add 0.0000000000000001 to the first key $K_{1}=\left(a,X\left(0\right)\right)$, we will not obtain the original image after decryption by using that key. It is clearly observed that any clue or gesture about the original image is not found in the encrypted image. The algorithms of our proposed cryptosystem are highly sensitive to secret keys.

### 4.12. Cryptanalysis

The cryptanalysts usually mount the chosen-plaintext attack and the chosen-ciphertext attack on a cryptographic technique to break it. By employing these types of attacks, many cryptographic techniques are cracked. We implement these types of attacks and show the resistance of our proposal against them.

#### 4.12.1. Chosen-Plaintext Attack

In this scenario of attack, the cryptanalyst has a ciphered image, but the encryption key is unknown. However, he has a plain image $P^{0}$ of all-zero (or all-one) and its corresponding ciphered image$\;C^{0}\;$obtained with the same unknown key. The cryptanalyst develops the following sub-key extraction for pixel encryption [19].

(13) $$SK_{i,j}^{0}=C_{i,j}^{0}⊕P_{i,j}^{0}$$

where $P_{i,j}^{0}$ is a null image in terms of grey values, $C_{i,j}^{0}$ is its corresponding encrypted variant, and (i,j) is the two-dimensional pixel position. Equation (13) gives a key stream$\;SK_{i,j}^{0}$. In trying to obtain the plain image $P_{i,j}$ of the ciphered one$\;C_{i,j}$, the cryptanalyst makes use of the key stream$\;SK_{i,j}^{0}$ as follows.

(14) $$P_{i,j}=C_{i,j}⊕SK_{i,j}^{0}$$

**Figure 10:**
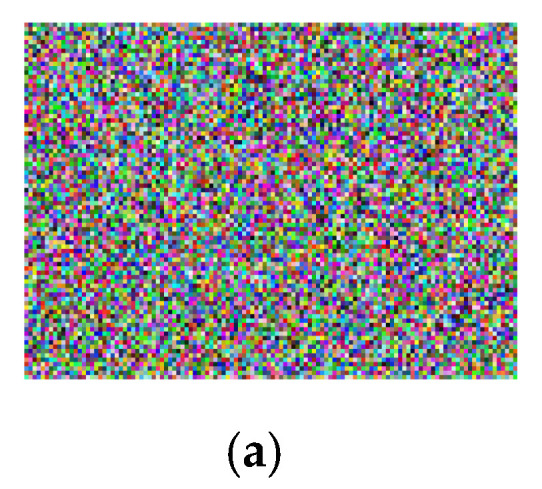

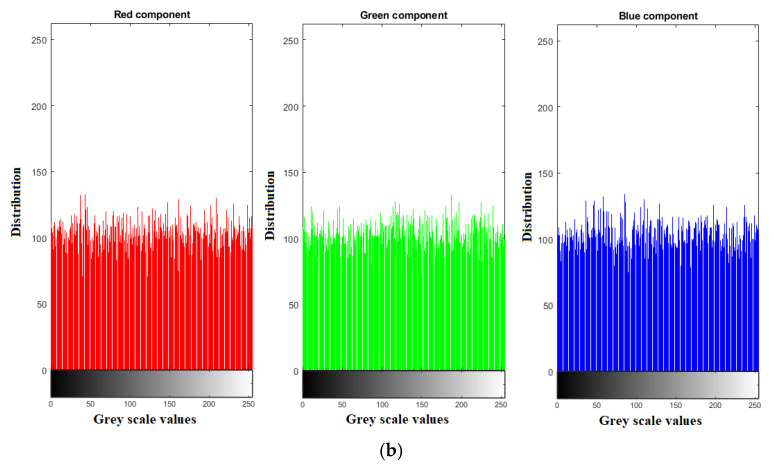
Cryptanalysis (**a**) chosen-plaintext attack of Lena image, (**b**) corresponding image histograms of Lena encrypted image (**a**).

In Figure 10a, it can be seen that the chosen-plaintext attack on the Lena encrypted image using a null image has failed. The corresponding histograms are given in Figure 10b. It is evident that the chosen-plaintext cannot be mounted in this proposed image encryption procedure. The reason for this failure is that pixel permutation and pixel diffusion phases rely on the techniques which are highly sensitive to insignificant change of a grey value. Therefore, the proposed technique demonstrates a strong resistance to the chosen-plaintext attack.

#### 4.12.2. Chosen-Ciphertext Attack

This is another type of attack having no information about the key. Knowing a ciphertext $C^{\prime }$ of all-one (or all-zero), and its corresponding decrypted variant $\;P^{\prime }$, the cryptanalyst tries to determine the key stream $K_{i,j}^{\prime }$ using Equation (13). Then, the plaintext $P_{i,j}$ would be acquired by Equation (14) [19].

**Figure 11:**
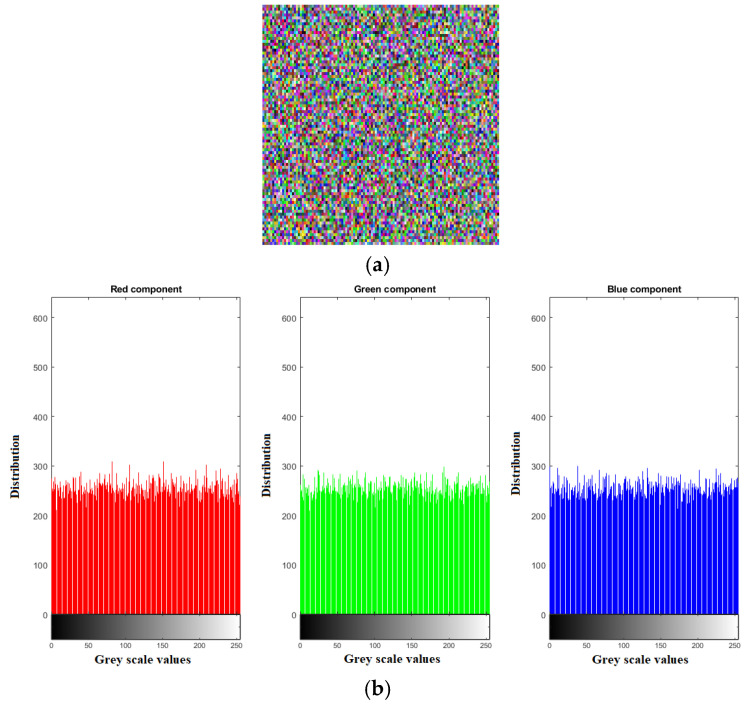
Cryptanalysis (**a**) chosen-ciphertext attack of Lena image, (**b**) corresponding image histograms of Lena encrypted image (**a**).

In Figure 11, the chosen-ciphertext attack on the Lena encrypted image with the null-images (all-zero pixel values) is shown. By observing the chosen-ciphertext attack of the Lena image and its corresponding histograms, it is evident that the chosen-ciphertext cannot be mounted in this proposed image encryption procedure.

## 5. Conclusions

In our paper, we proposed a novel image encryption technique using chaotic maps. The proposed technique first uses a Henon chaotic map to create a permutation phase. For substitution purposes, a Hill cipher is used whose key is generated from an orthogonal matrix by considering the equation of a plane. Then, in the next diffusion phase, a tent chaotic map is employed to obtain a sequence, and each pixel value is bitwise XORed with the values of the obtained sequence. The proposed algorithm works in two phases that are: the confusion phase is carried by Henon map and the diffusion phase is carried by chaotic tent map. The proposed algorithm has offered resistance to many cryptographic attacks, such as brute force attack. Security analysis is also conducted by using key space analysis, key sensitivity analysis, and entropy analysis. Security analysis tests of the method showed ascendancy on the security and authenticity of the Lena and onion images.

## Figures and Tables

**Table 1:** Comparison of Lena image histogram variance results.

Image	[12]	[13]	Proposed
Lena 256	982.5703	1071.0	980.50

**Table 2:** Comparison of chi-square test ($\chi_{t}{}^{2}$ test) results.

Image	Ref. [12]	Ref. [13]	Proposed	Testing Results	
				$\chi_{255,\;0.05}^{2}=$ 293.2478	$\chi_{255,\;0.01}^{2}$= 310.457
Lena 256	245.6426	267.7480	255.79	pass	pass

**Table 3:** Lena 256 correlation coefficient values.

Direction\Color	Red		Green		Blue	
	Original	Cipher	Original	Cipher	Original	Cipher
Horizontal	0.9794	0.0004	0.9806	−0.0013	0.9604	0.0073
Vertical	0.9574	−0.0028	0.9593	−0.0062	0.9237	−0.0014
Diagonal	0.9363	−0.0048	0.9400	−0.0002	0.8898	0.0064

**Table 4:** Performance of MSE.

Image Encryption Scheme	MSE
Ref. [12]	7762.6
Ref. [13]	7764.3
Proposed Scheme	8783.6

**Table 5:** Correlation coefficient values of onion (198 × 135) cipher image.

Directions\Colors	Red	Green	Blue
Horizontal	0.0091	−0.0051	0.0095
Vertical	0.0078	−0.0047	0.0093
Diagonal	0.0091	0.0152	−0.0056

**Table 6:** Comparison of NPCR and UACI values.

Image Encryption Schemes	NPCR	UACI
Ref. [14]	99.61	33.46
Ref. [15]	99.61	33.48
Ref. [16]	99.59	33.90
Ref. [17]	99.61	33.47
Standard values	99.61	33.46
Proposed scheme	99.61	33.46

**Table 7:** Comparison of entropy values.

Image Encryption Schemes	Entropy Values
Ref. [14]	7.9990
Ref. [16]	7.9967
Ref. [13]	7.9994
Proposed scheme	7.9992

## Data Availability

Data are available within the manuscript.
